# Inferring Microbial Interactions in the Gut of the Hong Kong Whipping Frog (*Polypedates megacephalus*) and a Validation Using Probiotics

**DOI:** 10.3389/fmicb.2017.00525

**Published:** 2017-03-30

**Authors:** Francis Cheng-Hsuan Weng, Grace Tzun-Wen Shaw, Chieh-Yin Weng, Yi-Ju Yang, Daryi Wang

**Affiliations:** ^1^Biodiversity Research Center, Academia SinicaTaipei, Taiwan; ^2^Department of Life Science, National Taiwan Normal UniversityTaipei, Taiwan; ^3^Biodiversity Program, Taiwan International Graduate Program, Academia Sinica and National Taiwan Normal UniversityTaipei, Taiwan; ^4^Department of Natural Resources and Environmental Studies, National Dong Hwa UniversityHualien, Taiwan

**Keywords:** network, gut microbiota, artificial hibernation, probiotics, *Polypedates megacephalus*

## Abstract

The concerted activity of intestinal microbes is crucial to the health and development of their host organisms. Investigation of microbial interactions in the gut should deepen our understanding of how these micro-ecosystems function. Due to advances in Next Generation Sequencing (NGS) technologies, various bioinformatic strategies have been proposed to investigate these microbial interactions. However, due to the complexity of the intestinal microbial community and difficulties in monitoring their interactions, at present there is a gap between the theory and biological application. In order to construct and validate microbial relationships, we first induce a community shift from simple to complex by manipulating artificial hibernation (AH) in the treefrog *Polypedates megacephalus*. To monitor community growth and microbial interactions, we further performed a time-course screen using a 16S rRNA amplicon approach and a Lotka-Volterra model. Lotka-Volterra models, also known as *predator–prey equations*, predict the dynamics of microbial communities and how communities are structured and sustained. An interaction network of gut microbiota at the genus level in the treefrog was constructed using Metagenomic Microbial Interaction Simulator (MetaMIS) package. The interaction network obtained had 1,568 commensal, 1,737 amensal, 3,777 mutual, and 3,232 competitive relationships, e.g., *Lactococcus garvieae* has a commensal relationship with *Corynebacterium variabile*. To validate the interacting relationships, the gut microbe composition was analyzed after probiotic trials using single strain (*L. garvieae, C. variabile*, and *Bacillus coagulans*, respectively) and a combination of *L. garvieae, C. variabile*, and *B. coagulans*, because of the cooperative relationship among their respective genera identified in the interaction network. After a 2 week trial, we found via 16S rRNA amplicon analysis that the combination of cooperative microbes yielded significantly higher probiotic concentrations than single strains, and the immune response (interleukin-10 expression) also significantly changed in a manner consistent with improved probiotic effects. By taking advantage of microbial community shift from simple to complex, we thus constructed a reliable microbial interaction network, and validated it using probiotic strains as a test system.

## Introduction

Gut microbes and their hosts exist in a symbiotic relationship. Gut microbes contribute to important host functions, including fermenting unused energy substrates, training the immune system, preventing growth of pathogenic bacteria, and regulating gut development (Hooper et al., 2002; Xu and Gordon, 2003; Li et al., 2008; Perez et al., 2010; Ye et al., 2014). Although recent studies using 16S rRNA amplicon sequencing have emphasized the importance of microbes for their hosts (Manichanh et al., 2006; Peterson et al., 2009; Round and Mazmanian, 2009; Turnbaugh and Gordon, 2009; Turnbaugh et al., 2009; Arumugam et al., 2011), the functional roles of most gut microbes remain unknown. One approach to explore this question is through network inference. This approach has been widely used to explore interactions between various organisms (Newman, 2003; Proulx et al., 2005; Shafiei et al., 2014). To uncover hidden patterns beyond the animal world, the generalized Lotka-Volterra (gLV) equations have recently been adopted as a dynamic model for studying microbial communities (Faust and Raes, 2012; Stein et al., 2013). The gLV model uses non-linear differential equations, which govern prey-predator relations. The gLV equations have been used successfully to predict temporal dynamics of microbiota in the mouse intestine (Stein et al., 2013), and within a cheese-making environment (Mounier et al., 2008), by analyzing microbiome time-series data. Time-series data inherently contain information including the statistical dependency of observations as a function of time. When these features of time-series data are properly modeled, it is possible to gain substantial new insights into the behavior of the system under study. Some studies even suggested that the distribution of interaction pairs (also obtained using a gLV dynamic model) in an ecological system can be used to predict microbiota stability (Coyte et al., 2015).

Network inference methods are commonly distinguished into two groups. The first approach is similarity-based network inference, which assesses the co-occurrence and/or mutual exclusion pattern of two species over multiple samples, using a measure that quantifies the similarity of two species' distributions. However, pairwise relationships do not capture more complex forms of ecological interactions, in which one species is influenced by (or depends on) multiple other species (Faust and Raes, 2012). To infer these types of interactions, the second approach is to apply regression-based networks, in which the abundance of one species is predicted from the combined abundances of other organisms. The latter is more convincing and was used by Stein et al. (2013) to reanalyze the *Clostridium difficile* infection data generated by Buffie et al. (2012). Marino et al. (2014) also used the gLV equations to model population dynamics of the gut microbiota in mice. However, these works generated interaction networks but did not further validate the inferred relations. To explore how the biological outcomes are directly related to the specific inferred microbial interactions, a well-investigated *in vivo* system describing the effects of gut microbes on their host must be applied for validation. Conventionally, the prevention and control of aquaculture diseases has focused on the use of vaccines or antibiotics (Pasteris et al., 2009b). However, treating or feeding frogs with antibiotics may cause the development of resistant bacteria (Akinbowale et al., 2006). Further evidence has shown that antibiotics can cause a decrease in the biodiversity of gut bacteria and increase the risk of bacterial infections (Buffie et al., 2012; Taur et al., 2012). Antibiotics and their effects can also persist for several days after the end of treatment (Jernberg et al., 2007; Dethlefsen and Relman, 2011; Buffie et al., 2012). An alternative solution is the use of probiotics (Reid et al., 2003), which are able to inhibit gut colonization by pathogens and to exert inhibitory effects against undesired micro-organisms, as well as to support natural host microbial defense mechanisms (Hernandez et al., 2005). Thus, a wide range of Gram (+) and Gram (−) bacteria, yeast, microalgae, and bacteriophages have been evaluated as probiotics (Pasteris et al., 2009b).

In this study, we highlight the feasibility of conducting network inference at the genus level to decipher the possible interactions within the microbial ecosystem of the amphibian gut. We took advantage of a well-investigated probiotic system to validate the inferred microbial interactions. Compared to the conventional strategy that orally introduces specific single bacterial species as probiotics, we emphasize the power of inferred network by simultaneously applying interacting microbial partners that can be used together to enhance the growth and beneficial effects of target probiotics. In addition to the validation of the inferred network, this test is also the first attempt to use a combination of cooperative strains as probiotics in raniculture.

The innate immune system is the first line of an organism's defense against infection. Probiotics interact with immune cells, such as mono-nuclear phagocytes, polymorphonuclear leukocytes, and natural killer cells, to enhance innate immune responses. Studies have shown that probiotics can increase the numbers of erythrocytes, granulocytes, macrophages, and lymphocytes (Balcazar et al., 2006; Akhter et al., 2015). The most commonly used probiotics in amphibians are lactic acid bacteria (LAB). LAB produce a range of important molecules such as organic acids, hydrogen peroxide, diacetyl, antimicrobial peptides (AMPs), and bacteriocins (Verschuere et al., 2000; Küng et al., 2014). The characterization of these compounds explains the beneficial effects of LAB; thus, some bacterial species with similar functions have been introduced as probiotics to restore beneficial microbial populations (Balcázar et al., 2007a; Pasteris et al., 2009b; Ringø et al., 2010; Mendoza et al., 2012). In this study, we induced a community shift from simple to complex via artificial hibernation (AH) in the treefrog and performed 16S rRNA amplicon analysis on the gut microbiome. Through the use of MetaMIS, a package that employs the gLV model to infer microbial interactions (Shaw et al., 2016), we generated the microbial interaction data and constructed the interaction network. In order to validate the microbial interactions, instead of the conventional strategy that only introduces bacteria with similar functions as probiotics, we selected a target LAB, and selected bacteria of the genera *Corynebacterium* and *Bacillus* as functional partners based on the network analysis. Our results showed that the combination of three representatives of these genera, *L. garvieae, C. variabile*, and *B. coagulans*, works more efficiently than any single strain, reflecting the reliability of the inferred microbial interaction.

## Materials and methods

### Sample collection

Eighty adult treefrogs (*Polypedates megacephalus*) were collected from New Taipei City and Taichung City, Taiwan. All animals were housed in 240-l glass tanks at 23°C under a 8:16 h light:dark cycle. Turkestan cockroach nymphs (Finke, 2013) were fed to the treefrogs at a quantity of 10% of treefrog biomass twice a week. The treefrogs were acclimatized for 3 months prior to experiments.

### Microbiome 16S rRNA amplicon analysis

Fecal samples were collected from treefrog guts within 20 min after euthanasia. To avoid cross-contamination, each sample was collected using a fresh pair of sterile tweezers. The contents of each gut were emptied into a sterile vial and immediately stored at –80°C. DNA was subsequently extracted with the QIAamp DNA Stool Mini Kit (Qiagen, Valencia, CA, USA). The V4 region (292 bp) of 16S rRNA gene was PCR-amplified with 515F (5′−GTGCCAGCMGCCGCGGTAA−3′) and 12-base barcoded 806R (5′−GGACTACHVGGGTWTCTAAT−3′) primers (Caporaso et al., 2012). Following PCR, samples were gel extracted with the NucleoSpin Gel Extraction kit (Macherey-Nagel, Germany). The purified samples were pooled in equal concentrations and sequenced using an Illumina MiSeq (Illumina, San Diego, CA, USA) with a V2 PE500 cartridge (500 cycles). All datasets have been deposited in GenBank under the BioProject ID PRJNA341914 and BioSample ID SAMN05730167 and SAMN05730170.

All paired-end sequences were merged by FLASH (Magoč and Salzberg, 2011), and all merged sequences were further analyzed by conducting the Quantitative Insights Into Microbial Ecology (QIIME) pipeline (Caporaso et al., 2010b). In the QIIME analysis pipeline, the low-quality sequences (sequences that were <200 bp in length, had a quality score <25, contained ambiguous characters, had an unreadable barcode, or did not contain the primer sequence) were removed using the USEARCH quality filter (Edgar, 2010). The UCLUST (Edgar, 2010) function in QIIME was used to cluster the remaining sequences, with a minimum coverage of 99% and minimum sequence identity of 97%. The longest sequences from each phylotype were selected to perform sequence identification, and PyNAST (Caporaso et al., 2010a) and UCLUST were selected to perform the sequence alignment and taxonomy assignment.

### Microbial community shift from simple to complex and the generalized Lotka-Volterra model

A concept for improvement of the investigation of the functional roles of gut microbiota is to explore the microbial world from a simple to complex state, from an initial stage to homeostasis. Hibernation is considered as a survival strategy designed to conserve energy when conditions are harsh. During hibernation, animals can adapt to temperature perturbations and extend their lifespan by slowing their heartbeat, reducing metabolic activity and their energy requirements (Book, 1974). At the same time, most microorganisms in the gut of a host decline in numbers due to extreme temperature and low nutrient supply (Gossling et al., 1982a). Hibernation of treefrogs, therefore, provides a natural model for monitoring microbial growth starting from inoculation (i.e., the beginning of a developing microbial interaction network) to homeostasis, and could provide an opportunity to shed light on our understanding of how microorganisms form their interaction networks.

To stimulate AH, we modified a program previously described in leopard frogs (*Rana pipiens*) (Gossling et al., 1982a). Fasting is known to reduce microbial complexity in amphibians, as well as fish, reptiles, birds, and mammals (Gossling et al., 1982b; Sonoyama et al., 2009; Costello et al., 2010; Kohl et al., 2014). Therefore, prior to stimulation of AH, food was withheld from the treefrogs kept in the same housing for 7 days to reduce overall diversity. All fasting treefrogs were then transferred into an incubator kept in constant darkness. AH was stimulated initially at 21°C with a relative humidity of 90%. During day 1, the temperature was maintained. During days 2–3, the temperature was gradually reduced to 4°C. Thereafter, treefrogs were housed in the same manner for 7 days. During days 11–12 (i.e., after 7 days of AH described above), the temperature was gradually increased to 21°C, and the treefrogs returned to their active status. Each active treefrog was fed with Turkestan cockroach nymphs (~0.25–0.3 g). Post-feeding samples were collected within 2.5 days. All protocols were approved by the Academia Sinica Biosafety Committee and Institutional Animal Care and Utilization Committee.

To collect time series samples for generating the microbial interaction network, we collected the gut contents of the treefrogs over a total of 12 time points spanning 15 days over the AH period, including day 1 (fasting for 7 days before AH), day 11–11.75 (every 6 h), and days 12–15 (every 12 h). For each time point, three to five treefrogs were euthanized. Details of treefrog body mass, sampling size, and time points of fecal collection over the AH period are presented in Supplementary Table 1.

We used the Metagenomic Microbial Interaction Simulator (MetaMIS) with a user friendly interface (Shaw et al., 2016) to infer microbial relations by introducing a single time-series dataset of microbial composition containing 12 time points. MetaMIS is a tool based on the generalized Lotka-Volterra model (Bhargava, 1989), and designed to infer underlying microbial interactions according to metagenomic abundance profiles. Lotka-Volterra equations have been widely used to infer animal interactions in dynamic systems, and recently have been applied to reveal microbial interacting relationships between operational taxonomic units (OTUs). The detailed algorithms and equations were described by Bucci et al. (Stein et al., 2013). Due to the limitation of computing power (Interl® Core™ i7-4770 CPU @3.40 GHz processor and 32 Gb RAM), it is not feasible to infer interaction network at the species level. Therefore, we assumed that in general, all species in a genus identified in our amplicon study share similar functions. According to the compositional profiles among 12 time points at the genus level, MetaMIS can systematically examine interaction patterns, such as mutualism or competition; only the top 25% of interacting relationships were considered in this study. We used the mfinder tool (Milo et al., 2002) to identify significant 3-node directed motifs that contain two directed edges pointing to the same node. This process was repeated 100 times and all relationships that passed the criteria described above at least 50 times (permutation cutoff > 0.5) were considered to be reliable interacting relationships.

### Probiotic selection and culturing

There are several genera of bacteria characterized as probiotic in amphibians (Pasteris et al., 2009a,b; Dias et al., 2010; Becker et al., 2011; Mendoza et al., 2012; Bletz et al., 2013). LAB have been considered as the major probiotics for the treatment of raniculture (Dias et al., 2010; Mendoza et al., 2012). In this study, *L. garvieae* was selected as a probiotic strain because *Lactococcus* was present in at least 85% of all the samples with a relative abundance of 0.14 ± 0.39%, and *L. garvieae* has been identified as a biological control agent in bullfrogs (Mendoza et al., 2012). According to the interacting relationships inferred by MetaMIS, *Corynebacterium* showed beneficial effects on *Lactococcus*, while *Bacillus* was found to have a beneficial interacting relationship with *Corynebacterium*. We decided to use three representative species of the three mentioned genera for further validation. Therefore, *L. garvieae, C. variabile*, and *B. coagulans* were chosen to validate the inferred interacting relationships. These three species were selected by their abundance ranking within the genus according to 16S rRNA amplicon data. We reasoned that if the inferred interacting relations were correct, the cooperative combination would yield a higher probiotic concentration than using a single strain.

To prepare for oral administration, *L. garvieae* and *B. coagulans* were grown in Tryptic Soy broth at 30 and 55°C respectively. *C. variabile* was grown in Brain Heart Infusion broth at 30°C. All strains were grown overnight with agitation in a shaking incubator. After incubation, the cells were harvested by centrifugation at 2,500 × g for 20 min at 4°C. The cell pellets were washed twice with 0.9% saline and resuspended using the same buffer. The measured population level of bacteria in the test diet was 10^7^ CFU g^−1^ (colony-forming unit).

Treefrogs were divided into five groups (*N* = 8 per group), and acclimated for 1 week before the start of the trial. The trial was conducted for a 2-week period. Each test group of treefrogs was dosed with *L. garvieae* (G_1_), *C. variabile* (G_2_), or *B. coagulans* (G_3_) singly, or with a combination of the three strains (G_4_) once per day by direct oral gavage. The control (G_5_) was fed with 0.9% saline during the entire trial period. After the trial, four fecal samples were collected from four treefrogs in each group (Supplementary Table 2) for further 16S rRNA amplicon analysis, and the other four treefrogs were used to perform quantitative PCR for interleukin 10 (IL-10) expression as described in the following section.

### Quantitative real time PCR for IL-10

In order to test the immune response after oral administration with probiotic, the expression level of IL-10 was measured after LPS (lipopolysaccharide) stimulation (Qi et al., 2015). To characterize the change of treefrog IL-10 expression after LPS stimulation, four treefrogs in each group were injected intraperitoneally (i.p.) with LPS (150 μg/100 g body weight). Animals were anesthetized and euthanized 24 h after injection. All fecal contents were removed and the remaining tissue samples of gut were collected (weight range 0.03 to 0.08 g) (Supplementary Table 3). All tissue samples were homogenized and total RNA was extracted from homogenized samples using Trizol reagent (Invitrogen, USA), quantified using a Nanodrop–1000 spectrophotometer, and reverse transcribed into cDNA using the Superscript II reverse transcription system (Invitrogen, USA) according to the manufacturer's instructions. Quantitative real time PCR was performed using Power SYBR green PCR Mastermix (Applied Biosystems) on a real-time instrument (ABI mode 7300 Sequence Detector) in 96-well reaction plates. The reaction mixture included 10 ml of Power SYBR green PCR Mastermix, 1 μl of forward and reverse primer (10 μM each) and 1 ml of cDNA, and then brought up to a final total volume of 20 μl with ultra pure water. The sequence of IL-10 in *P. megacephalus* was described in previous study (Huang et al., 2016) and the forward and reverse primers were designed by NCBI Primer-BLAST (Ye et al., 2012). β-actin was used as a housekeeping control. The primer sequences for amplification of IL-10 and β-actin were as follows: (F, 5′-ACGACCCTGCTCACGTTATG-3; R, 5′-TCCGGGATGGAGTAAGAGGG-3′) and (F, 5′-GGTCGCCCAAGACATCAG-3; R, 5′-GCATACAGGGACAACACA-3′) (Hamdan et al., 2016), respectively. The relative expression of IL-10 in gut tissue samples was normalized to the expression of β−actin. The change of gene expression was expressed as fold change (log base 2) and calculated as described (Qi and Nie, 2008; Qi et al., 2010, 2015). A paired Student's *t*-test was applied to analyze the significance and fold change (log base 2), with a *p*-value less than 0.05 considered to be statistically significant.

### Statistical analysis

To estimate the change of microbial complexity throughout the AH period, alpha-diversity was determined using the Shannon index, Simpson index, and the Inversed Simpson index. To determine the differences in bacterial community composition during AH, we used the Bray-Curtis similarity index (a taxonomic metric), which provides a measure of phylogenetic distance between communities from individual samples (Lozupone et al., 2007). To test the differences in richness and phylogenetic indices between time points, Student's *t*-test was used to determine the significance (*p*-value < 0.05) between time points. For comparison between each bacterial challenge and controls, we determined the fold-change in relative abundance to demonstrate a response to the stimulus relative to the background. Fold-change significantly higher than 2 or smaller than 0.5 was considered to be relevant. Differences between the two groups were analyzed for significance (*p*-value < 0.05) by Wilcoxon's test.

## Results

**Figure 1:**
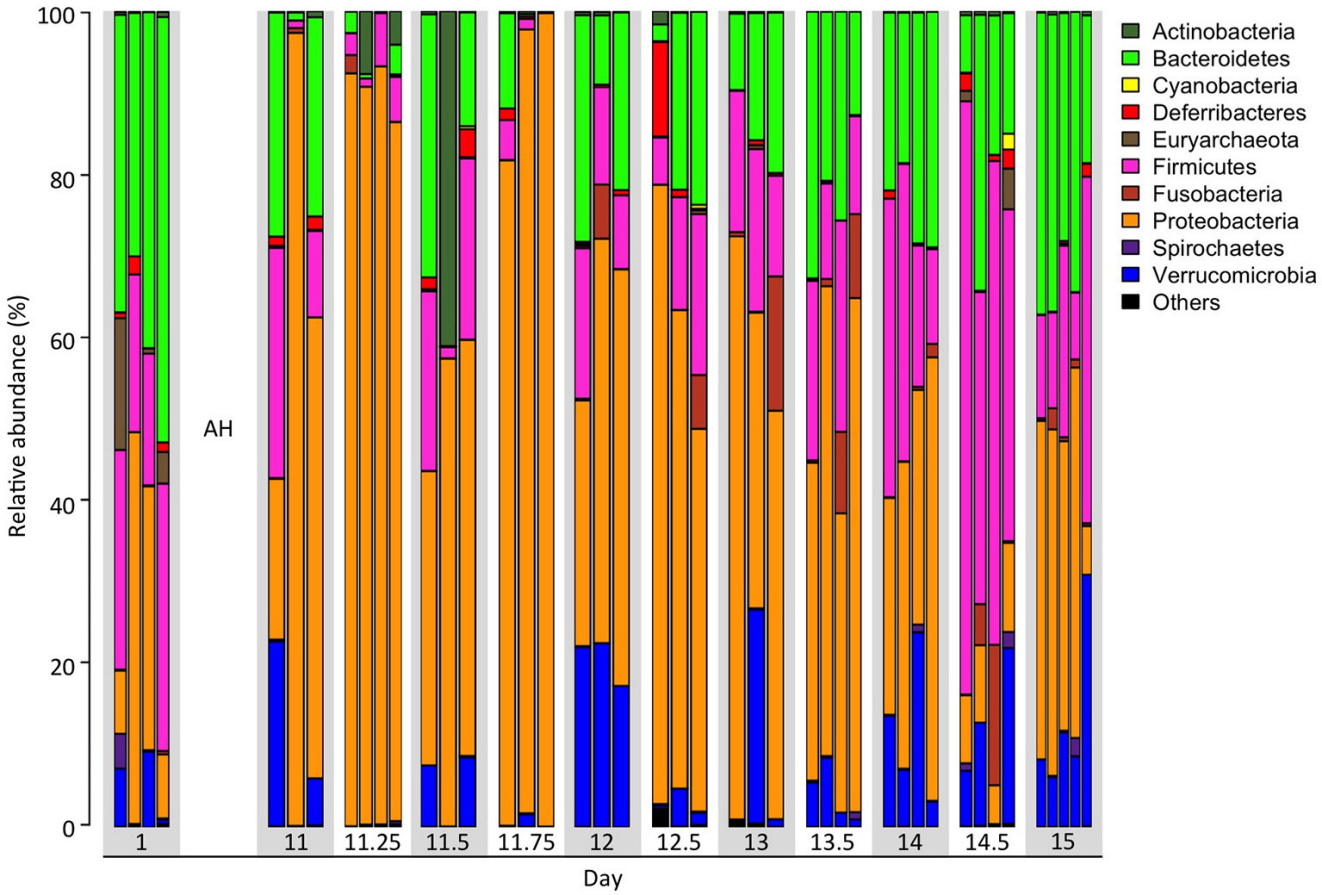
**Time dependent taxonomic composition spanning 15 days over AH**. Taxonomic composition of fecal microbiota over 12 time points including pre- and post-artificial hibernation (AH) at the phylum level. Each bar represents one individual.

In this study, we conducted a continuous 15-day time series data collection through communities from simple to complex to infer interacting relationships of microbes in the gut of treefrogs. We obtained an average of 128,608 ± 32,367 high quality, classifiable 16S rRNA gene sequences, with an average count per time point ranging from 91,908 ± 7,767 to 161,142 ± 45,301. At the numbers of reads generated for each sample, the numbers of genera were in the saturation phase (Supplementary Figure 1), indicating that genera from each sample had been sufficiently recovered in MiSeq sequencing. We observed that the phylogenetic indices, including the Shannon index and the Simpson Index, were significantly increased at all time points after day 12 (24 h after AH), compared with day 11.25 (6 h after AH). The Inverse Simpson Index was also significantly increased at days 12, 13.5, 14, 14.5, 15, compared with day 11.25 (Table 1). In addition, three predominant phyla were significantly altered in their relative abundance (Student's *t*-test, *p*-value < 0.05) from day 11.25 at all time points after day 13.5 (Figure 1). For example, the increased relative abundance of Bacteroidetes at days 13.5, 14, 14.5, and 15 were 23 ± 4.2%, 24 ± 2.5%, 18 ± 5.6%, and 31 ± 3.6%, compared with 1.7 ± 0.8% at day 11.25. The relative abundance of Firmicutes at day 11.25, 13.5, 14, 14.5, and 15 were 3.9 ± 1.3, 18 ± 3.6, 25 ± 6.5, 53 ± 8.2, and 20 ± 6.2%, while the relative abundance of Proteobacteria at day 11.25, 13.5, 14, 14.5, and 15 were 90 ± 1.6, 49 ± 6.6, 37 ± 6.3, 8.4 ± 1.3, and 34 ± 7.2%. We also found that other phyla were significantly increased, such as Tenericutes and Verrucomicrobia, or significantly decreased, such as Thermi, in relative abundance after day 13.5 compared to day 11.25. These observations suggest that AH successfully reduced the microbial complexity, which may enhance the accuracy of inferred interactions.

**Table 1:** **Time-dependent phylogenetic diversity spanning 15 days over AH**.

**Time (day)**	**Richness**	**Shannon index**	**Simpson index**	**Inverse Simpson index**
1	14 ± 1.47	1.32 ± 0.1	0.68 ± 0.03	3.17 ± 0.33
11	13.67 ± 1.45	0.94 ± 0.4	0.47 ± 0.21	2.56 ± 0.87
11.25	13.25 ± 0.25	0.41 ± 0.07^a^	0.18 ± 0.03^a^	1.22 ± 0.04^a^
11.5	12.67 ± 0.88	1.15 ± 0.2	0.63 ± 0.06^b^	2.81 ± 0.43
11.75	13.67 ± 0.67	0.3 ± 0.19	0.13 ± 0.09	1.18 ± 0.14
12	15 ± 1.15	1.36 ± 0.06^b^	0.69 ± 0.03^b^	3.32 ± 0.34^b^
12.5	17.33 ± 2.96	1.14 ± 0.12^b^	0.56 ± 0.08^b^	2.41 ± 0.42
13	19.33 ± 3.38	1.2 ± 0.17^b^	0.62 ± 0.09^b^	2.87 ± 0.57
13.5	11.75 ± 0.75	1.24 ± 0.05^b^	0.64 ± 0.04^b^	2.91 ± 0.31^b^
14	14 ± 0.41	1.32 ± 0.07^b^	0.69 ± 0.03^b^	3.36 ± 0.32^b^
14.5	14.25 ± 1.11	1.31 ± 0.14^b^	0.63 ± 0.07^b^	2.93 ± 0.49^b^
15	13.4 ± 1.21	1.31 ± 0.03^b^	0.68 ± 0.01^b^	3.17 ± 0.13^b^

*Within each column, values not sharing superscripts (a and b) differ significantly (p-value < 0.05, Student's t-test). Values are expressed as mean values ± SD*.

### The inferred interacting relations

To infer microbial interactions, we focused on the level of genera. Overall, 325 genera that were characterized via the QIIME pipeline were used to construct the interacting relationships network using MetaMIS. In each run, pairwise interacting relationships with defined criteria (such as commensalism or amensalism) were generated. We obtained interacting paired relationships for 26,320 to 26,327 pairs in 100 permutations. After analysis, 10,314 inferred interaction pairs (IIPs) passed the permutation criterion (cutoff > 0.5 described in Methods), including 1,568 commensal, 1,737 amensal, 3,777 mutual, and 3,232 competitive relationships (Supplementary Table 4). For reference, we also constructed the interaction network at the species level (Supplementary Table 5). Most inferred relations are consistent in both networks generated in the level of genera and species.

To choose probiotic bacteria for the validation of IIPs, we surveyed the literature and selected three genera, i.e., *Lactococcus, Lactobacillus*, and *Pediococcus* that have been commonly used in probiotic treatments in raniculture (Dias et al., 2010; Mendoza et al., 2012). In our dataset, there were 84, 64, and 60 IIPs correlated to *Lactococcus, Pediococcus*, and *Lactobacillus*, respectively. Therefore, we selected *Lactococcus* as the target probiotic. In order to select the commensal partners for *Lactococcus*, we further surveyed the genera that directly imposed beneficial effects on *Lactococcus* (inferred from the interaction network). *Corynebacterium* was selected due to the fact it had the highest beneficial effects in the network. Additionally, *Bacillus* imposed beneficial effects on *Corynebacterium* and was selected from 69 IIPs of *Corynebacterium*. Consequently, in this study, we performed oral administration using three representatives of these genera, i.e., *L. garvieae, C. variabile*, and *B. coagulans* to validate the inferred interacting relations in gut microbes of the treefrogs.

**Figure 2:**
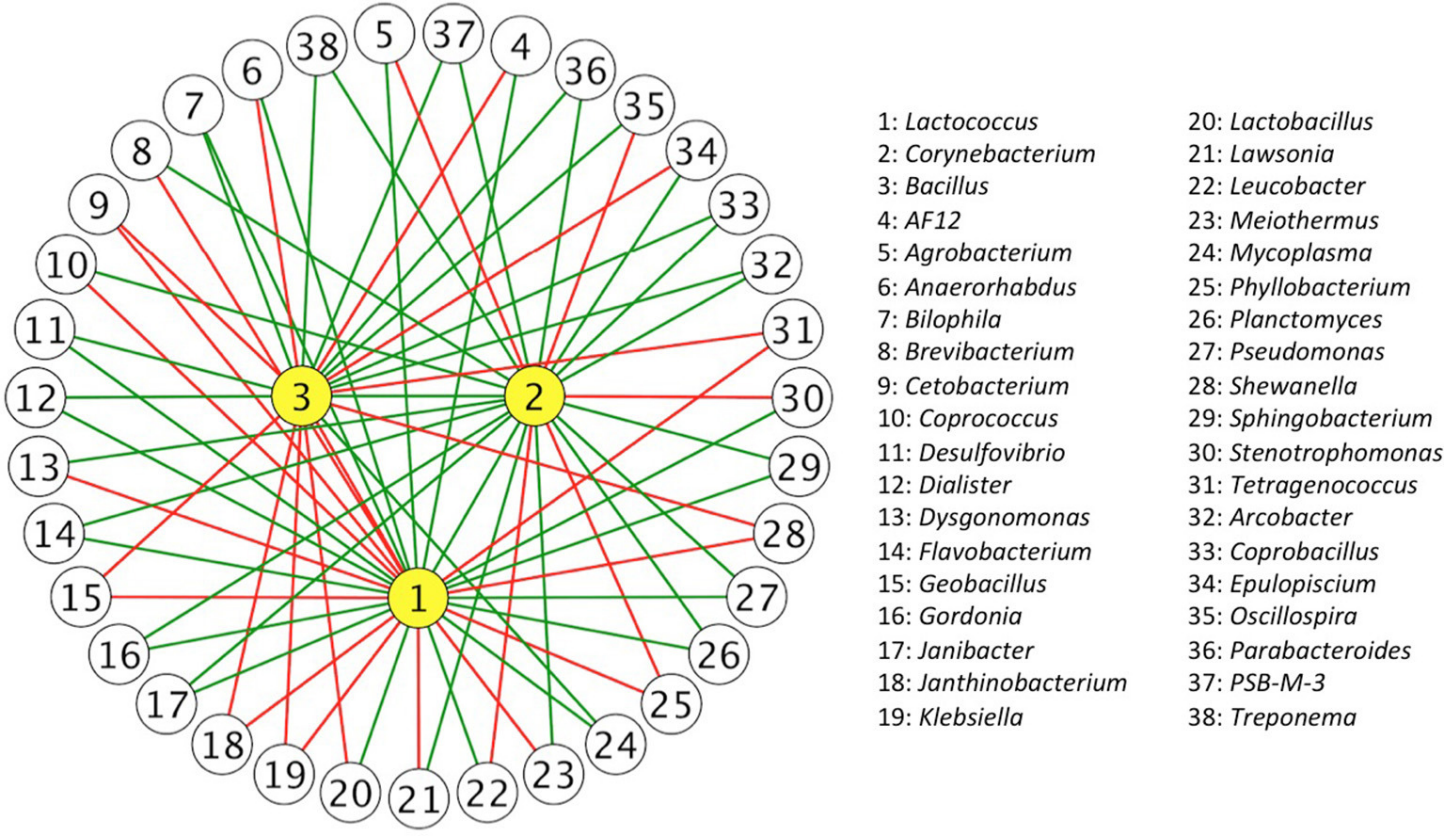
**Inferred interaction partners of ***Bacillus, Corynebacterium***, and ***Lactococcus*****. Complex relationships were inferred from gut bacterial communities in the 15-day time series data. Each node represents an inferred interaction partner (IIP) and each edge represents an inferred interaction relation between them. The edges in green represent commensal or mutual interactions, and the edges in red represent amensal or competitive interactions. Only the IIPs that contained two inferred interaction relationships are shown.

To further describe probiotic networks, we identified specific IIPs for *Lactococcus, Corynebacterium*, and *Bacillus* from 1,012 IIPs to pinpoint the genera that directly or indirectly interact with our selected bacteria (Supplementary Table 6). There were 84 IIPs interacting with *Lactococcus*, including one commensal, 41 mutual, one amensal, and 41 competitive. From the 84 interacting partners with *Lactococcus*, three of them were predominant genera (relative abundance on average was larger than 5%), *Bacteroides* (20.8 ± 16.2%), *Citrobacter* (6.6 ± 10.4%), and *Shewanella* (16.3 ± 24.1%), and were all inferred as competing partners, suggesting that a considerable population that colonized the treefrog intestine may intrinsically inhibit the genus *Lactococcus*. The number of IIPs with *Corynebacterium* and *Bacillus* were 69 and 60 respectively. It is worth noting that compared with *Lactococcus, Corynebacterium* had more mutual relations with other intestinal bacteria; there were no amensal relationships and only 21 of 69 IIPs were competitive. A small group of microbes that interact with *Lactococcus, Corynebacterium*, and *Bacillus* is shown in Figure 2.

### Oral administration of *L. garvieae, C. variabile*, and *B. coagulans*

**Figure 3:**
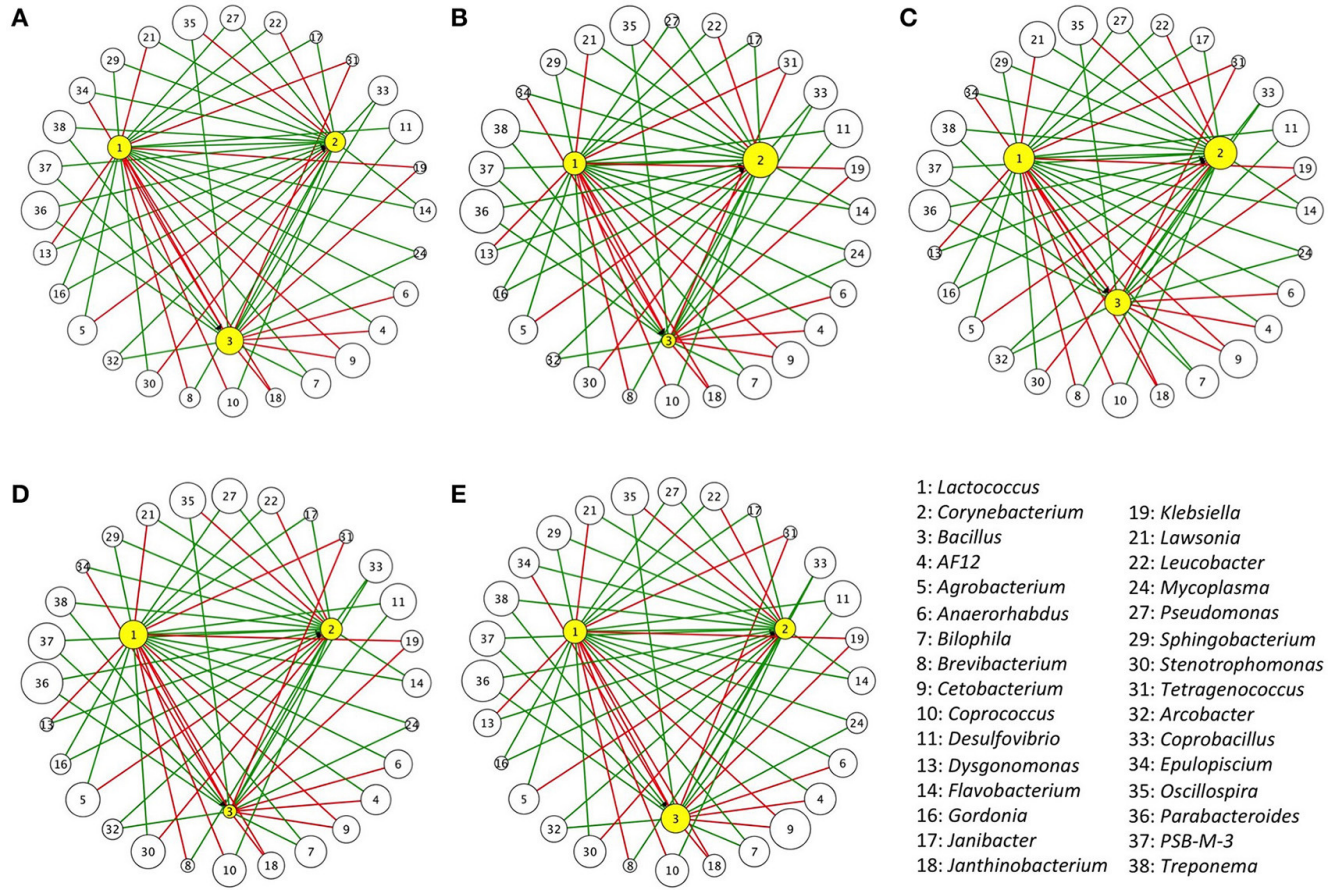
**Relative abundance of IIPs of ***Bacillus, Corynebacterium***, and ***Lactococcus*** after two-week oral trials**. Five oral administrations included **(A)** G_1_: *L. garvieae* (10^7^ CFU g^−1^) in 0.9% saline, **(B)** G_2_: *C. variabile* (10^7^ CFU g^−1^) in 0.9% saline, **(C)** G_3_: *B. coagulans* (10^7^ CFU g^−1^) in 0.9% saline, **(D)** G_4_: A combination of *B. coagulans, C. variabile*, and *L. garvieae* (each contains 10^7^ CFU g^−1^) in 0.9% saline, and **(E)** G_5_: Control (0.9% saline). Each node represents an IIP that is correlated with *Bacillus, Corynebacterium*, or *Lactococcus*, and each edge represents an inferred interaction relation between them. The edges in green represent commensal or mutual interactions, and the edges in red represent amensal or competitive interactions. To better visualize the distribution, the size of each node represents the relative abundance of gut microbes in logarithmic scale.

From the inferred interactions, 84, 69, and 60 IIPs were correlated to *Lactococcus, Corynebacterium*, and *Bacillus*, respectively. After oral administration of the three representative probiotics of these genera (*L. garvieae, C. variabile*, and *B. coagulans*), 65, 57, and 52 IIPs of *Lactococcus, Corynebacterium*, and *Bacillus* were identified in the trial by 16S rRNA amplicon analysis. The IIPs with abundance data of zero were excluded from the following analysis. Oral administration of three distinct bacterial species representative of each genus led to successful gut colonization, and led to a reasonable change in IIPs. In the G_1_ treatment group (oral administration of *L. garvieae*), *Lactococcus* significantly increased in relative abundance 9.88 ± 2.1-fold compared with control (G_5_ treatment group) (Supplementary Table 6). In the G_2_ treatment group (oral administration of *C. variabile), Corynebacterium* significantly increased in relative abundance 160.51 ± 58.54-fold (data insufficient to test the change of *Lactococcus*). On the other hand, in the G_3_ treatment group (oral administration of *B. coagulans)*, the relative abundance of *Bacillus* decreased 0.59 ± 0.49-fold, with no change in *Corynebacterium*, and a 1.91 ± 0.49-fold increase in *Lactococcus*. In the G_4_ treatment group (oral administration of a combination of *L. garvieae, C. variabile*, and *B. coagulans*), as expected, *Lactococcus* significantly increased in relative abundance 26.17 ± 6.96-fold (Wilcoxon's test, *p*-value < 0.05), 2.64 times greater increase than see for *L. garvieae* alone, reflecting the positive effects of the commensal partners. Also in the G_4_ treatment group, *Corynebacterium* increased 95.99 ± 9.57-fold in relative abundance (Wilcoxon's test, *p*-value = 0.0625), while *Bacillus* decreased 0.09 ± 0.04-fold in relative abundance in comparison with controls, reflecting the inhibitory effect of *Lactococcus*. Figure 3 and Supplementary Table 7 illustrated the relative abundances of microbes that interact with *Lactococcus, Corynebacterium*, and *Bacillus* after the two-week oral trials.

The inferred network describes the potential interactions among genera, in addition to the interactions mentioned above, more information remained to be discussed (Figure 3). For example, a recent *in vitro* experiment showed an inhibition of *L. garvieae* K2 against *Klebsiella pneumoniae* U11468 (Olaoye, 2016), and our network inference also suggests a similar interaction between these two species. To test the whole immune response after oral probiotic administration, IL-10, an immunoregulatory cytokine involved in immune response in amphibians, was used as an index. The frog IL-10 contains conserved amino acid residues and motifs that are essential for bioactivity. The same residues have been proved to be necessary for immunostimulatory function of human IL-10. Studies have been done using IL-10 expression to examine the host immune response to bacterial infection (Qi et al., 2015). In our study, we found that IL-10 expression variation was consistent with the level of *Lactococcus* in G_1_ to G_4_ treatment groups (Table 2). For example, IL-10 expression was increased in the G_1_ treatment group, while the corresponding level of *Lactococcus* in G_1_ was upregulated by 9.88, and the change was significant compared with controls (Wilcoxon's test, *p*-value < 0.05). In addition, IL-10 level in the G_4_ treatment group was significantly higher than in controls, also reflecting the high *Lactococcus* level in G_4_. These results also support the idea that a cooperative combination of *Lactococcus* triggered significantly higher expression of IL-10 than observed when using a single strain.

**Table 2:** **Expression analysis of IL-10 and level of ***Lactococcus*****.

**Treatment**	***N***	**Fold change of IL-10 (log base 2)**	**Fold change of *Lactococcus***
G_1_	4	1.64 ± 1.64	9.88 ± 2.1^*^
G_2_	4	1.48 ± 2.11	NA
G_3_	4	2.34 ± 1.81^*^	1.91 ± 0.49
G_4_	4	1.68 ± 1.22^*^	26.17 ± 6.96^*^

Gut tissues sampled for quantitative PCR analysis. The expression levels of IL-10 were determined relative to β-actin. The expression changes of IL-10 were expressed as fold change (log base 2) relative to controls. p-values generated by paired sample Student's t-test between test groups and controls are shown (

* *p-values < 0.05)*.

*The relative abundance changes of Lactococcus by 16S rRNA amplicon analysis were expressed as fold change relative to controls. Values are expressed as means ± SD. NA, data insufficient for test. L. garvieae (G_1_), C. variabile (G_2_), B. coagulans (G_3_), and a combination of the three strains (G_4_)*.

To validate the inferred interaction network, the corresponding changes of all IIPs were evaluated by calculating the fold changes between the test and control groups. In the G_1_ group, after oral administration of *L. garvieae* for 2 weeks, 52.3% (34 out of 65) of IIPs correlated with *Lactococcus* responded consistently with our inferred relations, including one commensal, 10 competitive, and 23 mutual interactions (Table 3 and Supplementary Table 6). There were 47.4% (27 out of 57) and 46.2% (24 out of 52) of IIPs correlated with *Corynebacterium* and *Bacillus*, respectively, which also responded consistently with our inferred relations. However, there were 23.1% (15 out of 65), 31.6% (18 out of 57), and 34.6% (18 out of 52) IIPs that correlated with *Lactococcus, Corynebacterium*, and *Bacillus*, presenting a conflicting response with our inferred relations, respectively. Overall, in the G_1_ treatment group, the ratio of IIPs that responded according to the inferred relationship was 48.9% (85 out of 174), the ratio of IIPs that showed a conflicting response to the inferred relationships was 29.3% (51 out of 174) (Table 3). In the evaluations for the G_2_, G_3_, and G_4_ treatment groups, we found 43.1–54.4% of corresponding changes of IIPs were consistent with the inferred interaction (Table 3).

**Table 3:** **Validation of IIPs that correlate with ***Lactococcus, Corynebacterium***, or ***Bacillus*****.

	**Total IIPs**	**Treatment**	**Consistent IIPs**	**Conflict IIPs**	**No difference**
*Lactococcus*	65	G_1_	34 (52.3%)	15 (23.1%)	16 (24.6%)
		G_2_	30 (46.2%)	14 (21.5%)	21 (32.3%)
		G_3_	28 (43.1%)	19 (29.2%)	18 (27.7%)
		G_4_	31 (47.7%)	16 (24.6%)	18 (27.7%)
*Corynebcaterium*	57	G_1_	27 (47.4%)	18 (31.6%)	12 (21.1%)
		G_2_	27 (47.4%)	13 (22.8%)	17 (29.8%)
		G_3_	31 (54.4%)	17 (29.8%)	9 (15.8%)
		G_4_	31 (54.4%)	13 (22.8%)	13 (22.8%)
*Bacillus*	52	G_1_	24 (46.2%)	18 (34.6%)	10 (19.2%)
		G_2_	24 (46.2%)	14 (26.9%)	14 (26.9%)
		G_3_	23 (44.2%)	19 (36.5%)	10 (19.2%)
		G_4_	27 (51.9%)	13 (25%)	12 (23.1%)

*Values are number of IIRs that correlate with Lactococcus, Corynebacterium, or Bacillus in each treatment group. The value in parentheses corresponds to the ratio of consistent IIPs, conflict IIPs, and no difference. L. garvieae (G_1_), C. variabile (G_2_), B. coagulans (G_3_), and a combination of the three strains (G_4_)*.

## Discussion

Recent advances in sequencing technology have created a new opportunity to explore population of microbes and their associations with environmental changes. Combining mathematical and computational models to infer the interacting networks reveals more details of microbe-microbe and microbe-host interactions. For example, Stein et al. (2013) extended generalized Lotka-Volterra equations to study the mechanism of *C. difficile* colonization in mice. They inferred that the genera *Akkermansia, Blautia*, and *Coprobacillus* had inhibitory interactions on *C. difficile*. In contrast, *Enterococcus* and *Mollicutes* could positively affect the growth of *C. difficile*, while the genus *Barnesiella* was predicted to inhibit growth of the genus *Enterococcus*. The results highlight a multi-layered sub-network associated with *C. difficile*. A study by Trosvik et al. (2010) also inferred microbial interactions in human infant gut using a dynamic systems modeling approach called time-dependent generalized additive models (GAM). They showed an agreement between predictions by dynamic interaction modeling and observed data of Firmicutes and Proteobacteria, suggesting that microbe-microbe interactions were sufficient to explain the growth patterns via modeling from time-series data. However, due to the complexity of microbial composition and difficulty in handling intestinal colonization, there was no experimental data to examine the inferred interactions (Mounier et al., 2008; Trosvik et al., 2010; Stein et al., 2013; Marino et al., 2014). To fill the gap, in this study, we used a user-friendly tool, MetaMIS (Shaw et al., 2016), to apply the Lotka-Volterra equations to infer the microbial interaction. The results were further validated by manipulating the gut bacteria and examine the corresponding changes in microbial composition.

Although MetaMIS is a versatile tool for predicting microbial relationships, Lotka-Volterra equations still have some weaknesses, for example, bias resulting from the complexity of microbiota. For data input, gLV equations require knowledge of the growth rates of all community members, and the complexity of microbiota may cause difficulties in determining the dynamic growth rate calculated from relative abundance, especially in the case of rare species (Pedrós-Alió, 2012). To improve the accuracy of MetaMIS, we implemented a time series data with the microbial communities from simple to complex for network inference. We firstly used AH in the treefrog to reduce the microbial diversity, and consequently less species were involved in the interaction network. After AH, increased temperature triggered bacterial turnover in composition, making it possible to reveal the evolving interactions between bacteria over time. The data also allowed us to collect time-series data after the perturbation to generate more dynamic fluctuations and provide deeper insights, compared with static communities (Holling, 1973; May, 1973, 1974; Ives and Carpenter, 2007). We therefore collected fecal samples in a 15-day time series, and increased the density of sampling points after AH to monitor dynamic changes. The experimental design of the data input could lead to a low ratio of inconsistent IIPs.

To validate the interactions, we focused on a small group of microbes that interact with the probiotic strains, which have been well-studied and applied in raniculture (Dias et al., 2010; Mendoza et al., 2012). The well-known LAB, *Bacillus, Enterococcus*, and *Lactococcus*, have been described and applied in many organisms (Balcázar et al., 2007b; Pasteris et al., 2009a; Dias et al., 2010; Nayak, 2010; Mendoza et al., 2012). Their inhibitory mechanisms against pathogenic bacteria have also been discussed (Hyronimus et al., 1998; Payot et al., 1999; Kesarcodi-Watson et al., 2008; Pasteris et al., 2009a). Our results indicated that the inferred network is biologically significant, and the compositional change of probiotics and the immune responses consistently supported our inference. In addition, our inferred relations were supported by the literature. For instance, *Enterococcus* competed with *Citrobacter* and *Staphylococcus* according to the inferred interaction, and these relationships were supported by previous studies showing that *Enterococcus* spp. 334 maintained its inhibitory effect against *Citrobacter freundii* and *Staphylococcus epidermidis* in *Lithobates catesbeianus* (Mendoza et al., 2012). Another evidence also showed that *Enterococcus faecium* imposed an inhibitory effect against *C. freundii* in *Rana catesbeiana* (Pasteris et al., 2009a). In addition, *L. garvieae* was found to inhibit *C. freundii* by the production of organic acid (Mendoza et al., 2012), and this correlation between *Citrobacter* and *Lactococcus* is in agreement with our IIPs. Furthermore, the inferred network suggested that *Corynebacterium* and *Lactococcus* were mutually beneficial partners. This is consistent with the culture-based studies showing that the culturable microbiota of milk consists of primarily of LAB such as *Enterococcus* and *Lactococcus*, and were often accompanied by the presence of *Corynebacterium* (Coppola et al., 2008).

In this study we generated a microbial interaction network of gut microbiota in the treefrog, and sub-groups of inferred relations were also validated. The experimental approach using probiotic administration indicated that the inferred interactions were reliable, and the results were also supported by the literature. However, there are still some puzzles remaining. For example, in the G_2_ treatment group, oral administration with *C. variabile* caused barely any beneficial effects on *Lactococcus* colonization. Although unexpected changes of gut probiotic levels were commonly observed after probiotic treatment (Hai, 2015; Ramos et al., 2015; Yang et al., 2015), we reasoned that more complex factors derived from other indirect IIPs also imposed various effects (commensalism, mutualism, amensalism, and competition) on these targets. The study is the first attempt to manipulate gut bacteria composition according to the inferred microbial interactions. We demonstrated the possibility that the gut microbiome can be changed accordingly. Moreover, our study provides a new potential strategy for the use of probiotics in raniculture.

## Author contributions

DW and FW designed the analyses. YY, CW, and FW collected the data. FW performed the analyses. GS implemented gLV model. DW and FW wrote the paper. DW was the principle investigator and conceived the analyses. All authors read and approved the final manuscript.

### Conflict of interest statement

The authors declare that the research was conducted in the absence of any commercial or financial relationships that could be construed as a potential conflict of interest.

## Supplementary material

Supplementary Figure 1 **Rarefaction analyses for the observed number of genera from 12 time points**. The rarefaction curves for each sample of 12 time points were displayed by different colors.

Supplementary Table 1 **Summary of sample information of 12 time points spanning 15 days over AH**. Values are expressed as mean values ± *SD*.

Supplementary Table 2 **Summary of sample information of oral administration for bacterial composition**. Oral administration of five treatments was carried out for 2 weeks. G_1_: *L. garvieae* (10^7^ CFU g^−1^) in 0.9% saline. G_2_: *C. variabile* (10^7^ CFU g^−1^) in 0.9% saline. G_3_: *B. coagulans* (10^7^ CFU g^−1^) in 0.9% saline. G_4_: A combination of *B. coagulans, C. variabile*, and *L. garvieae* (each contains 10^7^ CFU g^−1^) in 0.9% saline. G_5_:Control (0.9% saline). Values are expressed as mean ± *SD*.

Supplementary Table 3 **Summary of sample information of oral administration for quantitative PCR**. Oral administration of five treatments was performed over 2 weeks. G_1_: *L. garvieae* (10^7^ CFU g^−1^) in 0.9% saline. G_2_: *C. variabile* (10^7^ CFU g^−1^) in 0.9% saline. G_3_: *B. coagulans* (10^7^ CFU g^−1^) in 0.9% saline. G_4_: a combination of *B. coagulans, C. variabile*, and *L. garvieae* (each contains 10^7^ CFU g^−1^) in 0.9% saline. G_5_: Control (0.9% saline). Values are expressed as mean ± *SD*.

Supplementary Table 4 **Ten thousand and three hundred fourteen significant inferred interacting pairs (IIPs) identified by MetaMIS**. 10,314 IIPs passed the permutation criterion (cutoff > 0.5 described in Methods), including 1,568 commensal relationships (represented by O), 1,737 amensal relationships (represented by A), 3,777 mutual relationships (represented by M), and 3,232 competitive relationships (represented by C).

Supplementary Table 5 **The raw data for the interaction network at the species level**. 1,568 IIPs passed the permutation criterion (cutoff > 0.5 described in Methods), including 174 commensal relationships (represented by O), 211 amensal relationships (represented by A), 461 mutual relationships (represented by M), and 722 competitive relationships (represented by C).

Supplementary Table 6 **Fold change of the inferred relationships with ***Lactococcus, Corynebacterium***, and ***Bacillus*****. Commensalism is represented by O, amensalism by A, mutualism by M, and competition by C. Fold changes are expressed as mean values ± *SD*. NA, data insufficient for test.

Supplementary Table 7 **Relative abundance of IIPs of ***Bacillus, Corynebacterium***, and ***Lactococcus*** after 2-week oral trials**. Five oral administrations included G_1_: *L. garvieae* (10^7^ CFU g^−1^) in 0.9% saline. G_2_: *C. variabile* (10^7^ CFU g^−1^) in 0.9% saline. G_3_: *B. coagulans* (10^7^ CFU g^−1^) in 0.9% saline. G_4_: A combination of *B. coagulans, C. variabile*, and *L. garvieae* (each contains 10^7^ CFU g^−1^) in 0.9% saline. G_5_: Control (0.9% saline). Values are expressed as mean values ± *SD*.

## Abbreviations

IIP: inferred interaction partner
AH: artificial hibernation.
