# Supplementary figures and images for: Inferring Microbial Interactions in the Gut of the Hong Kong Whipping Frog (Polypedates megacephalus) and a Validation Using Probiotics
Source: Front Microbiol. 2017 Mar 30;8:525. doi: 10.3389/fmicb.2017.00525 (PMC5371668; doi:10.3389/fmicb.2017.00525)

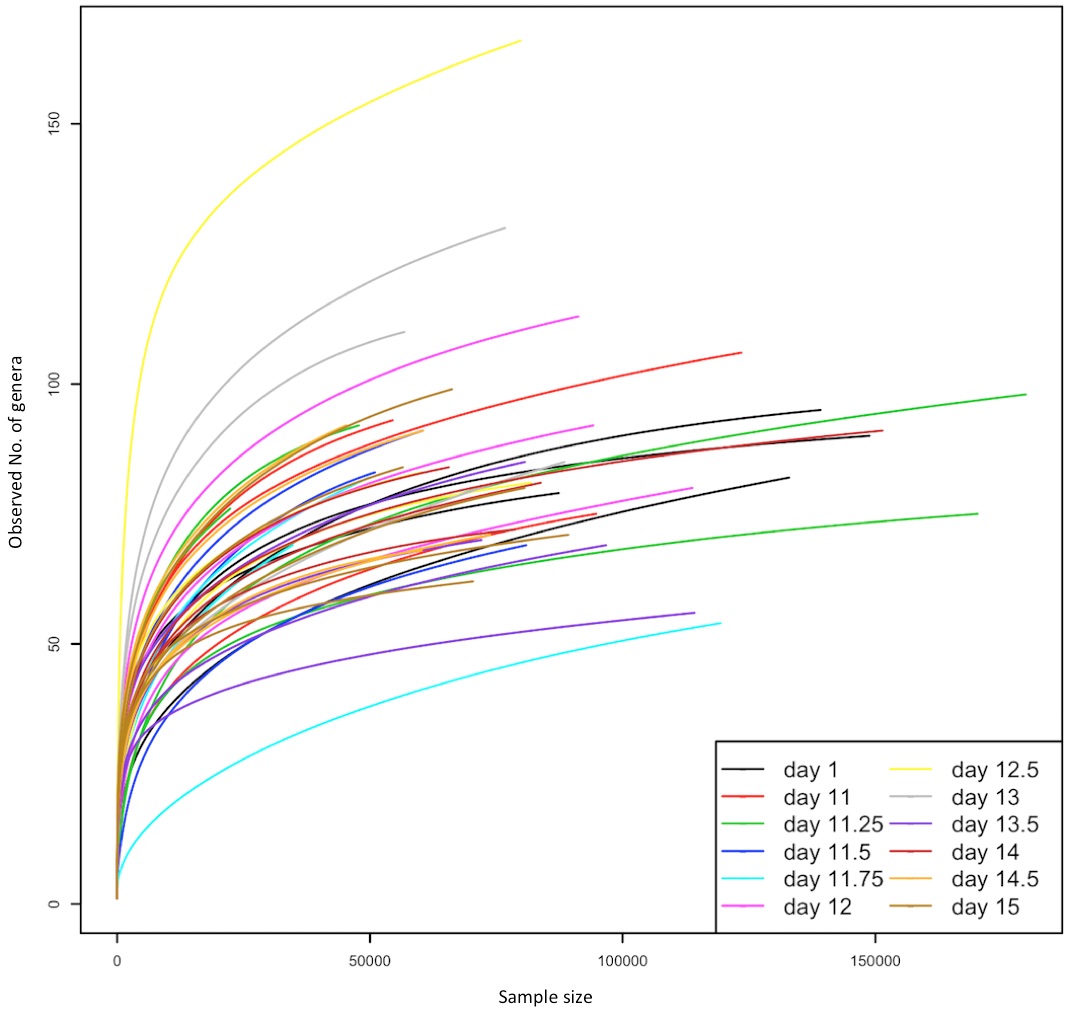

Supplement: Supplementary Figure 1 — Rarefaction analyses for the observed number of genera from 12 time points. The rarefaction curves for each sample of 12 time points were displayed by different colors. [file Image1.JPEG]
